# Hepatitis C virus management: potential impact of nanotechnology

**DOI:** 10.1186/s12985-017-0753-1

**Published:** 2017-05-02

**Authors:** Mostafa H. Elberry, Noureldien H. E. Darwish, Shaker A. Mousa

**Affiliations:** 1 The Pharmaceutical Research Institute, Albany College of Pharmacy and Health Sciences, Rensselaer, NY 12144 USA; 2 National Cancer Institute, Cairo University, Cairo, Egypt; 3 Faculty of Medicine, Mansoura University, Mansoura, Egypt

**Keywords:** Hepatitis C virus, Drug delivery system, HCV genotypes, Nanoparticles

## Abstract

Around 170–200 million individuals have hepatitis C virus (HCV), which represents ~ 3% of the world population, including ~ 3–5 million people in the USA. According to the WHO regional office in the Middle East, Egypt has the highest prevalence in the world, with 7% prevalence in adults. There had been no effective vaccine for HCV; a combination of PEG-Interferon and ribavirin for at least 48 weeks was the standard therapy, but it failed in more than 40% of the patients and has a high cost and serious side effects. The recent introduction of direct-acting antivirals (DAA) resulted in major advances toward the cure of HCV. However, relapse and reduced antiviral efficacy in fibrotic, cirrhotic HCV patients in addition to some undesired effects restrain the full potential of these combinations. There is a need for new approaches for the combinations of different DAA and their targeted delivery using novel nanotechnology approaches. In this review, the role of nanoparticles as a carrier for HCV vaccines, anti-HCV combinations, and their targeted delivery are discussed.

## Background

From the time of its discovery in 1989, hepatitis C virus (HCV) has been known globally as the primary reason for chronic liver disease [1]. HCV persistent infection is accompanied by liver cirrhosis, hepatocellular carcinoma, end stage liver disease and finally death [2]. Up to 3% of the world’s population, about 200 million individuals, is estimated to have HCV infection [3].

Among all the people infected with HCV, there are at least six genotypes [4] as a result of the high error rate of RNA-dependent RNA polymerases during HCV replication [5]. Genotype 1 (G1) is the most predominant in the world (~83.4 million people) followed by G3 (~54.3 million), then G2, G4 and G6 (~15.6 million), and G5 (~1.4 million) [6]. HCV transmission mainly occurs through contaminated blood and blood products transfusion, injection drug use, hemodialysis and organ transplantation; however unprotected sexual intercourse and birth from an infected mother have also been documented as other modes of transmission [7, 8].

Until 2011, the standard treatment was a blend of subcutaneous pegylated interferon (PEG-IFN) in addition to oral ribavirin (RBV), administered for 24 or 48 weeks [9]. The cure rate depends on the viral genotypes. IFN-alpha and RBV failed to eliminate HCV in 50–60% of patients infected with G1 and G4 and about 20% of patients with G2 and G3 [10]. Development of a new, specifically targeted antiviral therapy for HCV was a must to overcome the shortcomings of PEG-IFN/RBV therapy. This new targeted HCV therapy, called direct-acting antivirals (DAA), has been used in combination with PEG-IFN/RBV as a triple therapy since 2011. The first generation of protease inhibitors (PIs) DAA, telaprevir and boceprevir, was used in addition to PEG-IFN/RBV in the treatment of G1. They were associated with increased sustained virological response (SVR) rates up to 75% and 79%, respectively. Unfortunately they increased adverse effects and led to HCV drug-resistant mutants to both PIs [11–13]. Because of these drawbacks, a second wave of PIs is being developed, such as simeprevir and the second generation PI grazoprevir, in addition to nucleoside/nucleotide analogues like sofosbuvir and nonstructural protien 5A (NS5A) such as daclatasvir DAAs . These more efficient regimes are well-tolerated, pan-genotypic, and have a low tendency to develop viral resistance [14]. Along with DAAs, a number of host targeting antivirals (HTAs) have been developed. They act on host key enzymes or some cellular factors essential for the HCV life cycle [15]. The advantage of HTAs is that they have an extremely low rate of mutation in the host cell. The main targets of HTAs are cyclophilin A (CypA), miRNA, and fatty acid synthase.

**Fig. 1:**
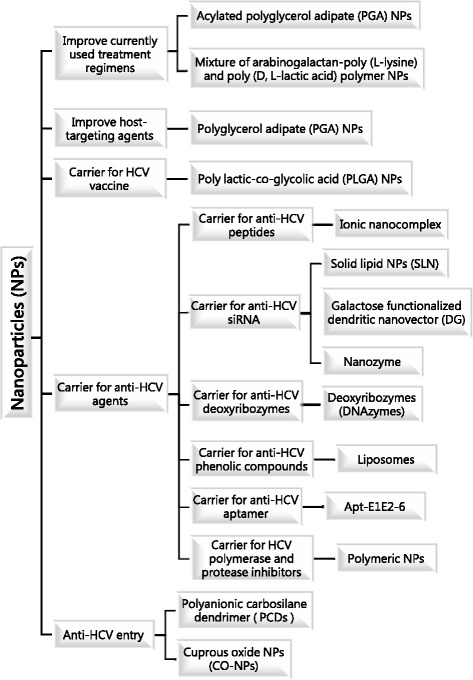
A summary diagram showing how nanoparticles are used as carriers for anti-HCV agents

The field of nanotechnology provides manipulation at the molecular level, and in the pharmaceutical field, nanomaterials have had a great impact on the enhancement of drug properties. Nanomaterials used in formulations can add high value due to their high dissolution rates, which can improve a drug’s bioavailability and stability. Biodegradable and biocompatible nanoparticles can be used as carriers for synthetic proteins, lipids, polysaccharides, and biopolymers [16]. Nanomaterials can also target specific organs, tissues, or even cells [17]. The introduction of anti-HCV targeted delivery using nanovectors, which carry the drug to its site of action, helps minimize the side effects of non-specific targeting and enhances therapeutic efficacy [18]. The aim of this review is to summarize new regimens for HCV treatment and the possible role of nanotechnology as a targeted delivery system that increases bioavailability and sustained drug release while decreasing severe side effects during HCV treatment. Techniques using nanoparticles to improve anti-HCV agents are listed in Table 1, and a summary diagram of nanoparticles used as carriers for anti-HCV agents is in Fig. 1.

**Table 1:** Advantages of using nanoparticles as a carrier for some anti-HCV compounds

Carrier for:	Composition	Advantages
RBV-boronic acid	PGA and acylated PGA NPs encapsulating RBV-boronic acid	Decreases RBV accumulation in red blood cells to help prevent hemolytic anemia
RBV monophosphate	Mixture of arabinogalactan–poly (L-lysine) and poly (D, L-lactic acid) polymer	1- Stable, biodegradable nanocomplex 2- Dual function of targeting hepatocytes and sustained release of RBV 3- RBV accumulates in liver of mice after i.v. administration of RBV monophosphate, then RBV content gradually decreases for at least 7 days
CsA	PLGA NPs	1- Reduces toxic effects associated with free CsA 2- Decreased immunosuppressive effects compared to conventional treatment with CsA
HCV vaccine	CpG oligodeoxynucleotide + recombinant HCV NS3 encapsulated in a cationic liposome	Increases not only cellular but also humoral immune response against HCV NS3
Anti-HCV peptides	P41, peptide derived from HCV NS5A + ionic nanocomplex	1- Decreases cytotoxicity, hemolytic effect and proteolytic degradation while maintaining antiviral activity against HCV 2- Inhibits HCV core and NS5A proteins from binding with lipid droplets, which is known to be an essential step for viral assembly and release
Anti-HCV siRNA	Galactose functionalized dendritic nanovector + siRNA against the 5' untranslated region of HCV genome	1- Improves cellular uptake of siRNA 2- Decreases rapid degradation of siRNA by nucleases and improves their blood stability
Anti-HCV deoxyribozymes	Iron oxide magnetic NPs as a carrier for DNAzyme Dz681	1- Inhibits HCV NS3 replication through the knockdown of HCV NS3 gene expression 2- Higher knockdown efficiency than free DNAzyme transfected with Lipofectamine 2000 3- Did not induce any undesired immune responses in vitro
Anti-HCV phenolic compounds	Silibinin, the active polyphenolic agent of milk thistle, incorporated with liposomes as a nanovector	1 -Improves solubility and delivery of silibinin 2 -Non-toxic and high antiviral activity to prevent entry with preferential absorption by hepatocyte
Anti-HCV aptamer	Magnetic nanoconjugate + aptamer (Apt-E1E2-6)	1- Efficiently eradicates HCV particles and decreases the viral titer from human plasma samples 2- Provides non-invasive technique for HCV removal with minimal side effects
HCV polymerase inhibitors and protease inhibitors	HCV protease and polymerase inhibitors + anti-fibrotic/anti-hemolytic + viral entry inhibitor agents + naturally driven polyphenol/thiols and non-anticoagulant GAGs	Allows for optimal antiviral efficacy and optimal safety profile

*Abbreviations*: *CsA* cyclosporine A, *NPs* nanoparticles, *PGA* polyglycerol adipate, *PLGA* poly lactic-co-glycolic acid, *RBV* ribavirin

## HCV virology

**Fig. 2:**
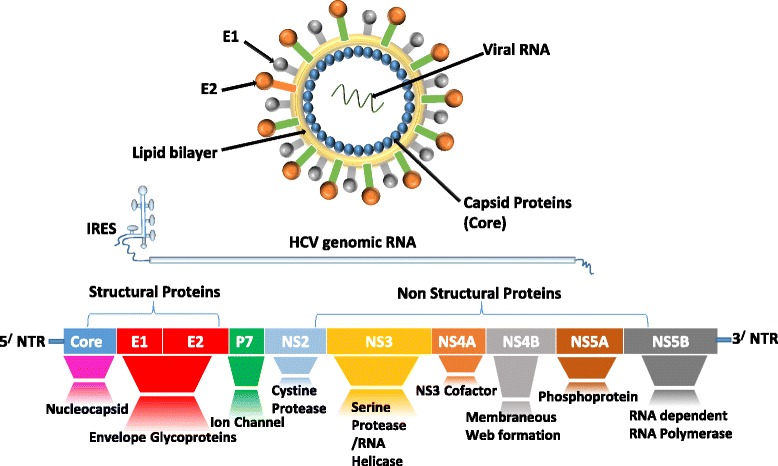
The HCV genome consists of a 9.6 kilobase ORF flanked with 5’ and 3’ untranslated regions. IRES-mediated translation of the ORF produces polyprotein that is processed by cellular and viral proteases into ten viral proteins: C, E1 and E2 structural proteins, and P7, NS2, NS3, NS4A, NS4B, NS5A and NS5B non-structural proteins. *Abbreviations*: C: Core protein, IRES: Internal ribosome entry site, ORF: Open reading frame, NS: Non-structural protein

HCV, one of the Flaviviridae virus family, is an enveloped positive single-stranded RNA virus. The HCV genome has 9600 bases, forming a continuous open reading frame edged by 5’ and 3’ non-translated regions [19]. The 5’ non-translated region contains an internal ribosome entry site (IRES) that is essential to initiate the translation of the HCV genome [20]. The IRES–mediated translation produces an approximately 3000 amino acid polyprotein precursor. It subsequently cleaves co- and post-translationally into mature viral structural and nonstructural (NS) proteins. The proteolytic processing of of the polyprotein is done by cellular peptidases [21] and two viral proteases, NS2/3 and NS3 [22, 23], which breaks it into 10 functional subunits: structural Core (C), envelope (E1 and E2) proteins, ion channel p7, and NS proteins NS2, NS3, NS4A, NS4B, NS5A and NS5B [24]. Subunits C, E1, and E2 form the virus particles in which nucleocapsid is constructed from repeated copies of the core protein, while E1 and E2 form the envelope glycoproteins. P7 may have a function in assembly and release of the viral particles. The NS proteins from NS3 to NS5B form the viral replicase complex. Also, NS5B forms the RNA-dependent RNA polymerase [25] (Fig. 2).

## New treatments for HCV

There are many FDA-approved anti-HCV therapies (Table 2), and some are still in different clinical trial phases.

**Table 2:** FDA-approved antiviral and combinations for HCV treatment in different genotypes

Genotype	Drug	PEG-IFN	RBV	Boceprevir	Telaprevir	Simeprevir	Sofosbuvir	Ledipasvir	Ombitasvir	Paritaprevir/ Ritonavir	Dasabuvir
*1*, *2*, *3*, *6*	PEG-IFN		●								
*1*, *2*, *3*, *6*	RBV	●									
*1*	Boceprevir	●	●								
*1*	Telaprevir	●	●								
*1*	Simeprevir	●	●								
*1*, *4*	Sofosbuvir	●	●								
*2*, *3*			●								
*1*			●			●					
*1*	Ledipasvir						●				
*1*	Ombitasvir									●	●
*1*			●							●	●
*4*	Paritaprevir/Ritonavir		●						●		
*3*	Dasabuvir						●				

*Abbreviations:* *PEG-IFN* pegylated interferon, *RBV* ribavirin

## Genotype 1

A recent phase II, open-label study assessed the use of daclatasvir (30 mg) plus simeprevir (150 mg) with or without RBV in G1 (a and b subtypes). For G1b treatment-naïve patients cirrhotic or non-cirrhotic, sustained virological response for 12 consecutive weeks (SVR12 rate) was 84.9% (45/53) and 74.5% (38/51) with and without RBV, respectively. For prior null responders it was 69.6% (16/23) and 95.0% (19/20) with and without RBV, respectively. In patients who completed 24 weeks, treatment duration had no significant impact on response; on the other hand, for G1a, the two DAAs with RBV provided a sustained virological response for 24 consecutive weeks (SVR24 rate) of 66.7% (8/12) in treatment-naïve patients, but they were not effective in prior null responders [26].

An open-label, phase II safety study of telaprevir and sofosbuvir in chronic hepatitis C G1 was conducted for 12 weeks on 20 non-cirrhotic and naïve HCV G1 infected patients who received telaprevir (1125 mg) twice per day and sofosbuvir (400 mg) once daily. The SVR12 rate after the end of treatment was 95%. The treatment was associated with some undesirable side effects such as a headache, pruritus, rash, and anorectal symptoms [27].

The OPTIMIST-1 open-label, phase III study of HCV G1 patients tested the efficacy and safety of simeprevir (150 mg) plus sofosbuvir (400 mg) for 8 or 12 weeks in both previously untreated and treatment-experienced non-cirrhotic patients. Results were an SVR rate of 83% in the 8-week treatment group, which was significantly lower than the SVR12 rate (97%) in the 12-week group. The study showed that the 8-week arm had high SVR12 rates only in subgroup 1b patients [28].

## Genotype 2

In a recent open-label, phase II study performed by a New Zealand team on 53 patients with HCV G2 (23% treatment-experienced and 4% with cirrhosis), sofosbuvir plus ledipasvir was administered for 8 or 12 weeks and achieved SVR rates of 74% and 96%, respectively. The most common side effects were headache, nausea, fatigue and upper respiratory tract infection [29, 30].

## Genotype 3

The ALLY3 phase III clinical trial studied the effectiveness of 12 weeks’ combined therapy of sofosbuvir (400 mg) and daclatasvir (60 mg) in 152 G3 infected patients. For both naïve (*n* = 101) and treatment-experienced (*n* = 51) patients, SVR12 rate was 90% (91/101) and 86% (44/51), respectively. It was distinctly lower in patients with cirrhosis (*n* = 32): 63% (20/32) vs. 96% (105/109) in non-cirrhotic patients [31].

## Genotype 4

In the PEARL-I phase 2b randomized, open-label trial, 12 weeks of ombitasvir (25 mg) plus paritaprevir (150 mg) plus ritonavir (100 mg) combination therapy (once daily), with or without RBV, was studied in 135 non-cirrhotic patients with chronic HCV G4. SVR12 rates of 100% (42/42) and 91% (41/44) were found in naïve patients with and without RBV, respectively, with no statistically significant differences. For previously treated patients, the SVR12 rate of the triple combination with RBV was 100% (49/49). No virological failures occurred in the RBV-containing regimen, while there was a 5% relapse and 2% virological breakthrough in naïve patients who did not receive RBV. There were some side effects such as headache, asthenia, fatigue, insomnia and anemia in some of the treatment-experienced patients on RBV [32].

The results from an open-labeled cohort, phase 2a trial that was conducted on HCV G4 naïve and treatment-experienced patients with or without cirrhosis who received sofosbuvir (400 mg) and ledipasvir (90 mg) combination for 12 weeks showed 95% (20/21) SVR12 rate. This study provided an IFN/RBV-free regimen for HCV G4 with a simple dosing of one tablet per day [33].

## Other genotypes

Across the world, HCV genotypes 5 and 6 represent only 1% and 5% of genotypes, respectively, from the total prevalence of all HCV genotypes, and the therapeutic trial for these genotypes has not been clear until recently [6].

## Pangenotypic

In a phase III trial (ASTRAL-1), the new DAAs regimen with velpatasvir (100 mg) and sofosbuvir (400 mg) once daily was evaluated in 624 patients across genotypes 1 to 6 in chronic HCV-infected patients, including those with compensated cirrhosis, for 12 weeks. The SVR rate was 99% for both treatment-naïve and treatment-experienced patients, including those with compensated cirrhosis. There were some serious adverse events in 2% of the patients such as extremity necrosis, acute myocardial infarction, appendicitis, cellulitis, chronic obstructive pulmonary disease, gastroenteritis, influenza, lung cancer, small intestinal obstruction, and sudden death from unknown cause and other symptoms [34].

In the COMMAND phase II study, patients with HCV G2 or G3 infection were treated with 60 mg daclatasvir plus PEG-IFN/RBV for 12 or 16 weeks compared to a PEG-IFN/RBV arm for 24 weeks. The SVR rate of 12- or 16-week treatment in HCV G2-naïve patients who received daclatasvir plus PEG-IFN/RBV was 83% in both durations compared to 63% in the PEG-IFN/RBV arm. In HCV G3 groups, the SVR24 rates after 12- or 16-week treatments were 69% and 67%, respectively, compared to the PEG-IFN/RBV arm (59%), so the addition of daclatasvir to PEG-IFN/RBV is associated with higher SVR24 rate compared with PEG-IFN/RBV alone in both genotypes. Moreover, daclatasvir plus PEG-IFN/RBV treatment had a tolerability and safety profile similar to that of PEG-IFN/RBV alone [35].

In a phase 3 study named C-EDGE, a 12-week course of grazoprevir 100 mg/elbasvir 50 mg was used in treatment-naïve patients with genotype 1, 4 or 6 among 421 cirrhotic and non-cirrhotic participants. Results from this study showed high SVR12 rates ranging from 80% up to 100% in treatment-naïve cirrhotic and non-cirrhotic patients according to the genotype. There were no serious treatment-related adverse events [36].

A study by Esteban and colleagues gave an option for genotype 2 and 3 patients who had experienced sofosbuvir failure. The patients received 400 mg sofosbuvir and 1000 mg RBV weekly for 24 weeks or sofosbuvir/PEG-IFN (180 mg)/RBV for 12 weeks. The overall SVR rates were 96% for G3 and 100% for G2 patients [37].

## MicroRNA as a therapeutic tool against HCV

MicroRNAs (miRNAs) are a class of short endogenous non-coding RNAs (~ 22 nucleotides) that mediate post-transcriptional regulation of gene expression [38]. They play important regulatory roles in many biological processes. miRNAs have emerged as a class of promising targets for HCV therapeutic intervention [38].

The important role of miR-122 as a host factor for HCV has been demonstrated. Binding of two miR-122 molecules with the 5′ untranslated region (UTR) of the HCV genome was found to protect the HCV 5′ UTR from nucleolytic degradation and thereby promoted viral RNA stability [39]. Moreover, miR-122 binding sites are conserved in all HCV genotypes. Locked nucleic acid-modified anti-miR miravirsen was recently reported with promising antiviral activity against all HCV genotypes [39, 40]. Miravirsen was initially assessed in an animal model for chronic HCV with 12 weeks of i.v. dosing [41]. The high dose treatment group (5 mg/kg) showed a marked and long-lasting decline of viral titer with no evidence of viral rebound or side effects in the treated animals. On the other hand, data from the first phase 2 study in HCV-infected patients showed a marked decline in HCV RNA levels (log10 IU/mL) in patients receiving 7 mg/kg [42]. Also in this study, no escape mutations were detected in the miR-122 binding sites, indicating the low incidence of miravirsen resistance.

## Potential of nanotechnology to improve currently used treatment regimens

Ribavirin is an effective nucleoside analogue used in the treatment of chronic HCV along with IFN. However, it has a restricted clinical application because it induces dose-dependent hemolytic anemia as a result of its accumulation inside red blood cells [43]. Many studies have reported the use of nanotechnology to try and solve this problem. For example, red blood cells have no endocytic mechanism [44] and low uptake of nanoparticles [45]. Polyglycerol adipate (PGA) and acylated PGA nanoparticles have been used as a carrier for RBV boronic acid, instead of using RBV only, because this provides RBV with hydrophobicity and increases the nanoparticles’ drug loading efficiency [46].

Ishihara et al. encapsulated RBV monophosphate in biodegradable nanoparticles prepared from a mixture of arabinogalactan–poly (L-lysine) and poly (D, L-lactic acid) polymer. The novel, stable, biodegradable nanocomplex showed a dual function of targeting hepatocytes and sustained release of RBV (37 days in PBS and 7 days after intravenous injection in mice). This nanoformulation is expected to display high antiviral activity and decrease the side effects of RBV [47].

## Potential of nanotechnology to improve host-targeting agents

Jyothi et al. used poly lactic-co-glycolic acid (PLGA) nanoparticles conjugated with liver targeting peptide (CKNEKKNKIERNNKLKQPP) to encapsulate cyclosporine A, an HCV inhibitor, via targeting the host factor cyclophilin A. These nanoparticles displayed high specificity to the liver cells, reduced the toxic and immunosuppressive effects of free cyclosporine A, and significantly inhibited HCV replication in a mouse model [48]. All of the above make this nanoformulation promising as a safe and efficient delivery system for HCV treatment.

## Nanoparticles as carrier for HCV vaccine

Jiao et al. utilized the ability of CpG oligodeoxynucleotide as an adjuvant and immunomodulatory together with recombinant HCV NS3 as an HCV vaccine model by encapsulating them in a cationic liposome. They found that this model increased not only cellular but also humoral immune response against HCV NS3. It dramatically induced a Th1 immune response against HCV [49].

In an animal study, mice were immunized with 10 mg recombinant HCV NS3 proteins with or without 50 mg CpG or GpC, either in free form or encapsulated in cationic liposomes. Samples were collected weekly for up to 8 weeks after immunization. Mice immunized with either liposome-NS3-CpG or liposome-NS3 produced 1:4-fold more cells secreting IFN-γ than secreted IL-4. The cells secreting IFN-γ in mice immunized with free-NS3 were only one-sixteenth of the cells secreting IL-4, indicating that liposomes encapsulating NS3 alone or with CpG have the ability to switch the immune response against HCV NS3 from the Th2 to the Th1 pathway [49].

## Nanoparticles as carrier for anti-HCV peptides

P41, an amphipathic alpha-helical cationic peptide derived from HCV NS5A membrane anchor domain C5A, was observed to have antiviral activity against HCV [50] and HIV [51], but rapid removal from circulation and toxicity of the cationic peptide restricted its use. So, Zhang et al. incorporated it into an ionic nanocopmlex that highly decreased cytotoxicity, hemolytic effect and proteolytic degradation while maintaining its antiviral activity against HIV and HCV [52].

Zhang et al. formulated an antiviral peptide nanocomplex using P41 electrostatically coupled with anionic poly (amino acid) based block copolymer with galactose as an active targeting moiety. This study revealed that galactosylated antiviral peptide nanocomplexes offered asialoglycoprotein receptor mediated uptake in hepatoma cell lines and robust inhibition of the HCV core and NS5A proteins from binding with lipid droplets, which is known to be an essential step for viral assembly and release [53].

## Nanoparticles as carrier for anti-HCV siRNA

RNA interference (RNAi) is a short interfering 21–23 nucleotides double-stranded RNA segment that can knockdown gene expression through managing mRNA degradation in a sequence-specific manner, and its mechanism depends on post-transcriptional gene silencing [54]. RNAi can precisely inhibit HCV RNA replication and protein expression in Huh-7 cells infected with HCV genome, and the antiviral effect is independent of IFN [55]. siRNA encounters some problems including low cellular uptake, rapid degradation by nucleases as well as inadequate blood stability [56], so many studies have used nanoparticles to solve these problems and to minimize the adverse effect of “off-targeting”. Lakshminarayanan et al. used a galactose functionalized dendritic nanovector (DG) as a carrier for siRNA against the 5' untranslated region of the HCV genome [18]. The siRNA-DG formed a stable complex that had target-oriented delivery through the interaction between its free galactose residues and asialoglycoprotein receptor. The delivered siRNA localized in the perinuclear region (the site of HCV replication) in which NS3 and NS5b viral proteins are co-localized. There was a significant inhibition in JFH1 RNA titer, so this new siRNA-DG nanovector is a promising option for HCV therapeutics [18].

Torrecilla et al. used an RNA interference strategy to inhibit the IRES by using shRNA74 short hairpin plasmid decorated on solid lipid nanoparticles (SLN) with hyaluronic acid (HA) or dextran (DX) as a non-viral vector. The SLN-HA and SLN-DX nanoparticles were shown to protect shRNA74 from DNase activity and rapid cell trafficking. The silencing efficiency of SLN-HA was more than SLN-DX [57].

Hang et al. found that cuprous oxide nanoparticles (CO-NP) at concentration of 2 μg/ml significantly inhibited the HCVcc/Huh7.5.1 cell line and also inhibited the HCVpp entry by more than 70%. This study revealed that CO-NPs blocked HCV attachment and entry. They hypothesized that CO-NP probably interacts with the surface of virion that in turn blocks the receptor binding sites present on the HCV envelope and thus prevent viral attachment [58].

## Nanoparticles as carrier for anti-HCV deoxyribozymes

Deoxyribozymes (DNAzymes) are RNA cleaving, short DNA molecules that can cleave a short target RNA substrate in a sequence-specific way [59]. They are tremendously efficient under simulated physiological environments, less expensive than siRNA, and can be easily chemically modified and with higher stability than RNA in serum [60]. Iron oxide magnetic nanoparticles (MNs) were used as a carrier for DNAzyme Dz681 selected for HCV NS3 RNA targeting in addition to cell penetrating peptide (MPAP) as an anti-HCV nanoformulation. Dz-MPAP-MN inhibited HCV NS3 replication in a Huh-7 Luc-Neo cell line through the knockdown of HCV NS3 gene expression. Moreover, it had a higher knockdown efficiency than the free DNAzyme transfected with Lipofectamine 2000 and did not induce any undesired immune responses in Huh-7 cells. Thus it has potential as a novel tool in clinical treatment of HCV [61, 62].

## Nanoparticles as carrier for anti-HCV phenolic compounds

Silibinin, the active polyphenolic agent of milk thistle, has been indicated to inhibit HCV virus entry and replication [63, 64], but it has poor water solubility and bioavailability. Ripoli et al. used liposomes as a nanovector to solubilize and deliver silibinin. The designed nanoparticles were found to be non-toxic and had high antiviral activity to prevent entry with preferential absorption by hepatocytes [65].

## Nanoparticles as carrier for anti-HCV aptamer

Aptamers are single-stranded oligonucleotide sequences, RNA or DNA, and can bind with high affinity and specificity to a wide range of targets such as peptides, proteins, drugs, and whole cells and viruses depending on their specific binding pockets for the target molecule. Nanomaterial-based aptamer bioconjugates have generated substantial interest and a wide variety of applications in medicine [66]. In an attempt to mechanically decrease the HCV load in plasma, Delaviz et al. designed a specific magnetic nanoconjugate for HCV treatment using an aptamer (Apt-E1E2-6) with a high affinity for HCV E1 E2 glycoproteins. The aptamer-conjugated magnetic nanoparticles efficiently eradicated HCV particles and decreased the viral titer from human plasma samples, providing a non-invasive technique for HCV removal with minimal side effects [67].

## Nanoparticles as carrier for HCV polymerase and protease inhibitors

A novel nanoformulation is a combination of HCV protease and polymerase inhibitors along with anti-fibrotic/anti-hemolytic and viral entry inhibitor agents using a combination of naturally driven polyphenol/thiols and non-anticoagulant GAGs [68]. These compounds are effective antiviral agents, especially in inhibiting the function of the various genotypes of HCV. Another polymeric nanoparticle-based technology platform incorporated the PI RBV or taribavirin and various types of polymerase inhibitors in the treatment of hepatitis C, along with anti-fibrotic/anti-hemolytic viral entry inhibitor [68, 69]. Furthermore, a conjugation with a therapeutic peptide, p14 (NS3 peptide) that confers the ability to target viral NS3 helicase is anticipated to increase the efficacy of the drugs encapsulated into the nanoparticle platform [69]. Drug-loaded nanoparticles were attached to monoclonal antibody fragments directed against epitopes conserved on the HCV surface E2 glycoprotein of genotypes 1a, 1b, 2a, 2b and 4 for targeted delivery of antiviral drugs [69]. Thus, the nano-incorporation of PIs and polymerase inhibitors along with anti-fibrotic/anti-hemolytic and viral entry inhibitors allow for optimal antiviral efficacy and optimal safety profiles [68, 69].

## Nanoparticles alone as an anti-HCV

Sepúlveda-Crespo et al. have identified a family of polyanionic carbosilane dendrimers that can inhibit HCV infection in tissue culture. The most effective of these compounds was G2-S24P (EC50 = 3 ± 1 nM). It inhibited HCV infection more than 80% by interfering with extracellular virions at the early steps of viral entry. It also displayed an additive effect in combination with sofosbuvir [70].

## Conclusions

In recent years many diverse scientific strategies have been developed for HCV therapy. Therapeutic options for HCV infection have been limited by drug resistance and adverse side effects. Targeting of the HCV replication was a promising strategy for antiviral therapy. However, due to its immunosuppressive activity and severe side effects, clinical applications in this category have been limited. To overcome these obstacles, researchers now have successfully developed drug delivery systems by conjugating the different antiviral drugs to different models of nanoparticles. Also, nanoparticles provide an alternate method for antigen delivery, which not only activates different elements of the immune system but also has good biocompatibility. One of the ways in which nanoparticles are able to elicit different immune responses is through their size; they move into cells via non-classical pathways and then are processed as such. One of the most widely used delivery systems is lipid nanoparticles. They are characterized by simplicity of production and safety, which encourages their use for in vivo application of RNAi. Moreover, lipid nanoparticles may be functionalized to reach target cells, and they may be used to combine RNAi molecules with conventional drug substances to reduce resistance or improve efficiency.

One of the unique advantages of nanotechnology in addition to the efficiency of drug delivery to pathological areas is its ability to decrease drugs’ toxicity and side effects. We herein discussed that a nanoparticle delivery system can be safe and may be a Trojan horse for future therapies against HCV.

## Abbreviations

DAA: Direct-acting antivirals
HCV: Hepatitis C virus
HTA: Host targeting antiviral
IRES: Internal ribosome entry site
PEG-IFN: Pegylated interferon
PI: Protease inhibitor
RBV: Ribavirin
SVR12: Sustained virological response for 12 consecutive weeks
SVR24: Sustained virological response for 24 consecutive weeks
